# Loss of HIF-1α in the Notochord Results in Cell Death and Complete Disappearance of the Nucleus Pulposus

**DOI:** 10.1371/journal.pone.0110768

**Published:** 2014-10-22

**Authors:** Christophe Merceron, Laura Mangiavini, Alexander Robling, Tremika LeShan Wilson, Amato J. Giaccia, Irving M. Shapiro, Ernestina Schipani, Makarand V. Risbud

**Affiliations:** 1 Department of Orthopaedic Surgery, Medical School, University of Michigan, Ann Arbor, Michigan, United States of America; 2 Inserm, UMRS 791-LIOAD, Centre for Osteoarticular and Dental Tissue Engineering, Group STEP ‘Skeletal Tissue Engineering and Physiopathology’, Nantes, France; 3 LUNAM, Nantes University, Faculty of Dental Surgery, Nantes, France; 4 Department of Orthopaedic and Traumatology, Milano-Bicocca University, Monza (MB), Italy; 5 School of Medicine, Department of Anatomy and Cell Biology, Indiana University, Indianapolis, Indiana, United States of America; 6 School of Medicine, Department of Radiation Oncology, Division of Radiation and Cancer Biology, Stanford University, Stanford, California, United States of America; 7 Department of Orthopaedic Surgery and Graduate Program in Cell and Developmental Biology, Thomas Jefferson University, Philadelphia, Pennsylvania, United States of America; 8 Department of Medicine, Division of Endocrinology, Medical School, University of Michigan, Ann Arbor, Michigan, United States of America; National Centre for Scientific Research, 'Demokritos', Greece

## Abstract

The intervertebral disc (IVD) is one of the largest avascular organs in vertebrates. The nucleus pulposus (NP), a highly hydrated and proteoglycan-enriched tissue, forms the inner portion of the IVD. The NP is surrounded by a multi-lamellar fibrocartilaginous structure, the annulus fibrosus (AF). This structure is covered superior and inferior side by cartilaginous endplates (CEP). The NP is a unique tissue within the IVD as it results from the differentiation of notochordal cells, whereas, AF and CEP derive from the sclerotome. The hypoxia inducible factor-1α (HIF-1α) is expressed in NP cells but its function in NP development and homeostasis is largely unknown. We thus conditionally deleted HIF-1α in notochordal cells and investigated how loss of this transcription factor impacts NP formation and homeostasis at E15.5, birth, 1 and 4 months of age, respectively. Histological analysis, cell lineage studies, and TUNEL assay were performed. Morphologic changes of the mutant NP cells were identified as early as E15.5, followed, postnatally, by the progressive disappearance and replacement of the NP with a novel tissue that resembles fibrocartilage. Notably, lineage studies and TUNEL assay unequivocally proved that NP cells did not transdifferentiate into chondrocyte-like cells but they rather underwent massive cell death, and were completely replaced by a cell population belonging to a lineage distinct from the notochordal one. Finally, to evaluate the functional consequences of HIF-1α deletion in the NP, biomechanical testing of mutant IVD was performed. Loss of the NP in mutant mice significantly reduced the IVD biomechanical properties by decreasing its ability to absorb mechanical stress. These findings are similar to the changes usually observed during human IVD degeneration. Our study thus demonstrates that HIF-1α is essential for NP development and homeostasis, and it raises the intriguing possibility that this transcription factor could be involved in IVD degeneration in humans.

## Introduction

The intervertebral discs (IVDs) are located in between each vertebra, and allow proper motion and adequate distribution of mechanical forces along the spine. The IVD is a complex anatomical structure made of three distinct tissues, namely the nucleus pulposus (NP), which occupies its inner portion, the annulus fibrosus (AF), which surrounds the NP, and the cartilaginous endplates (CEP) covering this assembly on both sides (top and bottom). The NP is a highly hydrated gelatinous tissue enriched in proteoglycans [1]. The AF is composed of 15–25 concentric fibrocartilaginous layers enriched in collagen fibers [2]. The CEP is a thin bilayer of hyaline cartilage and subchondral bone located between the vertebrae and IVD, and it is tightly anchored to the AF [3]. The NP is embryologically distinct from the other components of the IVD, as it is derived from the notochord whereas AF, CEP and vertebrae originate from the sclerotome [4]–[6].

The IVD is one of the largest avascular structures in vertebrate organisms. Recent studies have shown that the transcription factor HIF-1α (hypoxia inducible factor) is stabilized in the NP [7]–[10], most likely as an adaptive response to a variety of stresses including low oxygen tension [11]–[15]. Moreover, it has been demonstrated that activation of HIF-1α pathway has a critical role in a variety of NP cellular functions *in vitro* including, survival, proliferation, regulation of metabolism, and matrix synthesis [7]–[10]. Nevertheless, little is known about the putative role of HIF-1α in the changes occurring in the NP *in vivo* throughout embryological development, growth, aging and degeneration.

To understand whether and how HIF-1α affects NP biology *in vivo*, in this study we specifically deleted HIF-1α in notochordal cells, and we investigated NP development and homeostasis in mutant and control mice from E15.5 up to 4 months of age.

## Materials and Methods

### Generation of Mice

Generation and genotyping of the HIF-1α (FVB/N), Foxa2^iCre^ knock-in mice (FVB/N) and ROSA26 mT/mG reporter mice (FVB/N) have been previously described [16]–[19].

Foxa2^iCre^ knock-in male mice were bred with homozygous HIF-1α floxed (HIF-1α^f/f^) females, in order to obtain Foxa2^iCre^ positive heterozygous floxed (Foxa2^iCre^;HIF-1α^f/+^) male mice. These newly generated males were crossed with female mice homozygous for the floxed HIF-1α allele to generate Foxa2^iCre^;HIF-1α^f/f^ mutant mice, Foxa2^iCre^; HIF-1α^f/+^ and HIF-1α^f/f^ mice were used as control. We found that the Foxa2^iCre^ knock-in allele *per se* did not affect any of the phenotypes described in this manuscript. Generation of mT/mG reporter mice has been previously described [18].

### Animal euthanasia procedure

All procedures involving mice were performed in accordance with the NIH guidelines for use and care of live animals, and were approved by the University of Michigan Institutional Animal Care and Use Committee (IACUC permit number PRO00005182). For fetal and newborn time points, anesthesia was induced by hypothermia followed by decapitation.

For animals older than P10, anesthesia was induced by prolonged exposure to isoflurane followed by cervical dislocation; bilateral pneumothorax was also performed as a secondary method of euthanasia.

### Routine Histology, Safranin O Staining, Whole Mount Alizarin Red S/Alcian Blue Staining

For light microscopy, tissues from E13.5, E15.5 (delivered by caesarean section), newborn (NB), 1 and 4 month-old mice were fixed in 4% Paraformaldehyde (PFA)/Phosphate Buffer Saline (PBS) (pH 7.4) for 48 h at 4°C, and then stored in 70% ethanol at 4°C. NB and postnatal specimens were decalcified in 20% Ethylenediaminetetraacetic acid (EDTA) pH 7.5 at 4°C for up to 10 days. Paraffin blocks were prepared by standard histological procedures. Sections (5–6 µm thick) were cut from several levels of the block, and stained with Hematoxylin and Eosin (H&E). For Safranin-O staining, paraffin sections from E15.5 to 1 month-old spines were stained with Safranin-O/Fast green according to standard protocols [20]. Whole mount Alizarin Red S/Alcian Blue staining was performed as previously described [21].

### Cryosections

Specimens were dissected from E15.5 embryos and 1 month-old mice, fixed in 4% PFA/PBS at 4°C for 48 hour and then stored in 70% ethanol at 4°C. Specimens were subsequently placed in 30% Sucrose/PBS overnight and then embedded in optimum cutting temperature (OCT) embedding medium. Samples were sectioned at a thickness of 10 µm using a Leica cryostat. Sections were stored at −80°C for later use.

### In Situ Hybridization

*In situ* hybridizations were performed on paraffin sections from spines of E13.5 and E15.5 mice using complementary ^35^S-labeled riboprobes, as previously described [21]. The dual brightfield/darkfield technique allows detection of intense dark signal in brightfield and of weak bright signal in darkfield, respectively.

### TUNEL

TUNEL assay was performed on paraffin sections from spines of E15.5, NB, 1 month and 4 months mice using an "*In situ* cell death detection" Kit (Roche Diagnostic, Mannheim, Germany). Sections were permeabilized with 0.1% TritonX100 in 0.1% sodium citrate; TUNEL assay was then carried on according to manufacturer's instructions. Sections were then overlayed with Vectashield Hard Set mounting medium containing 4′, 6-diamidino-2-phenylindole (DAPI) (Vector Laboratories, Burlingame, CA, USA). Images were captured using filters for fluorescein isothiocyanate (FITC) and DAPI.

### BrdU Incorporation

E15.5 pregnant mice were injected intraperitoneally with 100 mg 5-bromo-2'-deoxyuridine (BrdU)/12 mg 5′-fluoro-2′-deoxy-uridine (FdU) per gram body weight 2 hours prior to sacrifice. After sacrifice, embryo spines were dissected, fixed, and embedded in paraffin, and longitudinal sections across the spine were obtained. To identify actively proliferating cells, nuclei that had incorporated BrdU were detected using a Zymed BrdU immunostaining kit (Invitrogen Corporation, Frederick, MD, USA). Both total number of cells and BrdU-positive cells where manually counted in the vertebral body (VB), in the AF and in the NP of the first lumbar intervertebral disc; proliferative rates were then calculated as number of BrdU-positive cells divided by total number of cells.

### Immunohistochemistry

For immunohistochemistry detection (IHC), paraffin sections from spines of E15.5 mice embryo were treated with sodium citrate buffer pH 6 at 95°C for 10 minutes. Sections were then incubated with the following primary antibody: anti-HIF-1α at a 1:100 dilution (LS-B495/46721, LifeSpan Biosciences) overnight at 4°C. After incubation with the appropriate biotinylated secondary antibody, detection of the binding was carried out using the labeled streptavidin biotin (TSA) system following manufacturer's instructions (Perkin Elmer, Shelton CT, USA). Negative controls, for HIF-1α IHC, have been performed by omitting the primary antibody.

### Image Acquisition

Images were acquired with Eclipse E800 (Nikon). Additional images were captured with a Leica DM LB compound microscope (Leica Microsystems). All the pictures were focused on the first lumbar intervertebral disc. For fluorescent images, frozen sections were dehydrated at 4°C overnight and wash in 1X PBS then overlayed with coverslips onto Vectashield Hard Set mounting medium (Vector Laboratories, Burlingame, CA, USA). Photos were taken using filters for Red Fluorescent Protein (RFP) and fluorescein isothiocyanate (FITC).

### X-rays imaging

Radiographic images were obtained using a Faxitron X-ray cabinet. Two month-old specimens were exposed for 8 seconds to a 20 keV radiation. Exposed films were developed in a dark room using a Hope MicroMax X ray processor (Hope X Ray Products Inc., Warminster PA) and scanned for quantitative analysis.

### Histomorphometry

IVD, VB, AF and NP delineation, area and thickness measurements have been performed using Photoshop CS6 (Adobe, San Jose, CA) and Bioquant Osteo 2014 (Bioquant, Nashville, TN) softwares in a blind randomized manner.

### Ex vivo spinal segment loading for intervertebral disc damping capacity

To evaluate the mechanical consequences of Foxa2-Cre-induced HIF-1α deletion on intervertebral discs, alterations in mechanical damping capacity of the intervertebral discs were measured via dynamic biomechanical tests. The 4^th^ to 5^th^ lumbar vertebral segment was carefully dissected from the spinal column of 12 freshly sacrificed mice (6 HIF-1α^f/f^ and 6 Foxa2^iCre^;HIF-1α^f/f^) without disturbing the intervertebral disc. The L_4_-L_5_ segment was cleaned of muscle and the neural arch was removed at the pedicles using dissecting scissors. The VBs were rinsed in saline and secured in custom grip fixtures (modified pin vises) that were mounted to a low-force materials testing instrument (Bose TestBench 3200). The vertebral segment was mounted such that one grip secured the L_4_ body, the other grip secured the L_5_ body, and the intervertebral disc remained unrestricted between the two fixtures. The spinal segments were loaded in oscillating tension and compression (±1.5 N) using 19 cycles of a sinusoidal waveform at increasing frequency. The bout began at 0.5 Hz, and then increased by 0.5 or 1 Hz after each successive load cycle until 15 Hz was reached. Force output from the load cell, displacement, and command signal were recorded for each test. Each segment was subjected to two rounds of testing.

Phase shift angles between applied sinusoidal forces and displacements of the spinal segments were determined. Force (*f*) and displacement (*L*) were modeled as a sinusoidal wave:

*(1) f* = *A_f_ sin* (ωt + *θ_f_*) + *B_f_*

*(2) L*  =  *A_L_ sin* (ωt + *θ_L_*) + *B_L_*

and the phase shift angle was determined as using a least square-mean fit method with MatLab software (version 7.10, MathWorks). In response to a sinusoidal force waveform at 0.5 Hz, 2 Hz, and 15 Hz, energy loss, Δ*E*, was determined as a hysteresis loop integral.

### Statistical analysis

All the experiments were performed using multiple sections of at least three independent biological replicates for each time point.

Results are represented as the mean of the replicates ± SD. Statistical differences were analyzed using the Student's t test. Differences with a *p*-value <0.05 were considered as statistically significant.

## Results

### Generation of mice lacking HIF-1α in the notochord

**Figure 1:**
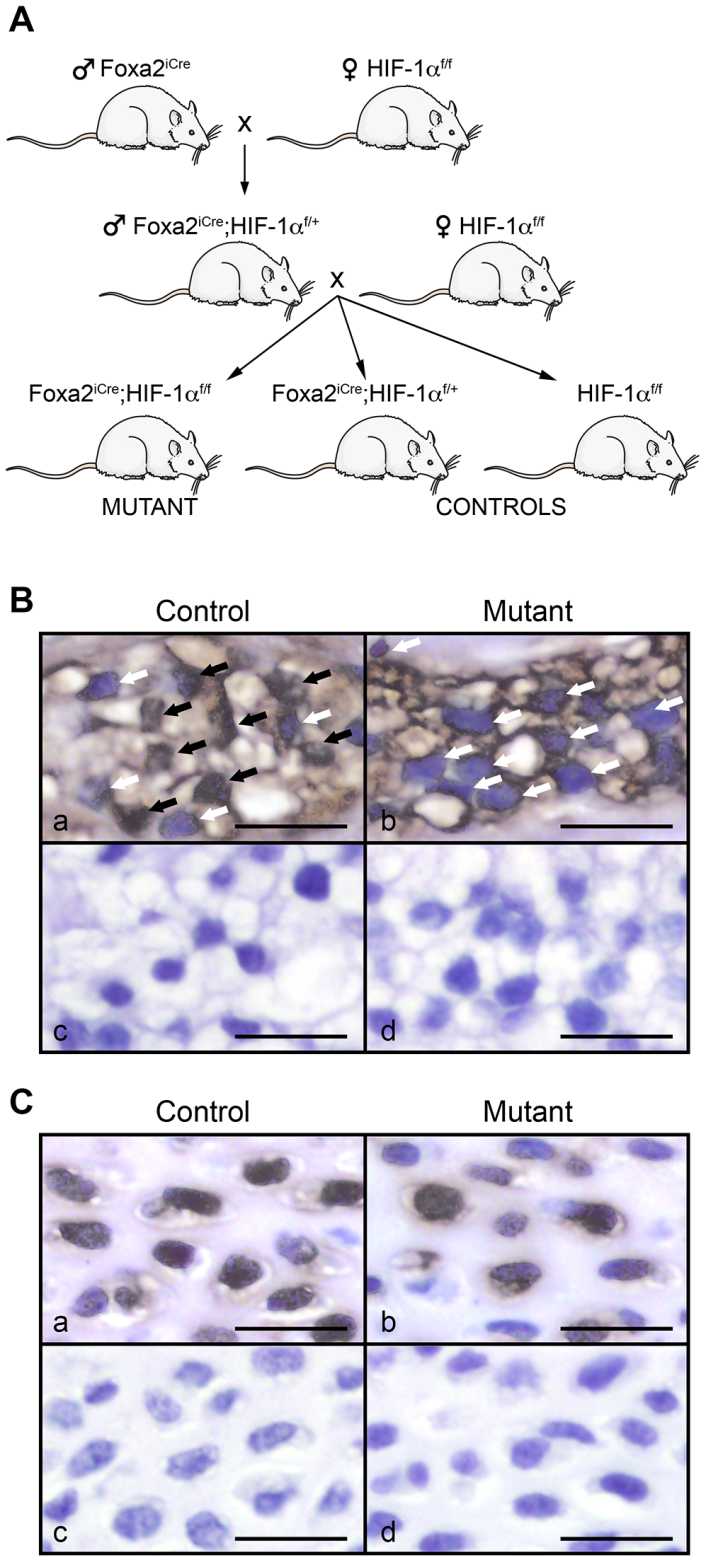
Specific and efficient deletion of HIF-1α in the nucleus pulposus of mutant mice. **A**. Breeding scheme used to generate Foxa2^iCre^;HIF-1α^f/f^ (mutant), Foxa2^iCre^;HIF-1α^f/+^ and HIF-1α^f/f^ (control) mice. **B**. HIF-1α immunohistochemistry in the nucleus pulposus of control (HIF-1α^f/f^) (a) and mutant (Foxa2^iCre^;HIF-1α^f/f^) (b) mice at E15.5. Black and white arrows indicate the presence and the absence of HIF-1α at the nuclear level, respectively. Respective negative controls for which the primary antibody has been omitted are shown (c and d). Bar = 50 µm. **C**. HIF-1α immunohistochemistry in the vertebral body of control (HIF-1α^f/f^) (a) and mutant (Foxa2^iCre^;HIF-1α^f/f^) (b) mice at E15.5. Respective negative controls for which the primary antibody has been omitted are shown (c and d). Bar = 50 µm.

We conditionally inactivated HIF-1α in the notochord by breeding Foxa2^iCre^ male mice with female mice homozygote for a floxed HIF-1α allele (HIF-1α^f/f^). Newly generated Foxa2^iCre^;HIF-1α^f/+^ male mice were bred with HIF-1α^f/f^ females in order to obtain Foxa2^iCre^;HIF-1α^f/f^ mutant mice, Foxa2^iCre^;HIF-1α^f/+^ and HIF-1α^f/f^ control littermates (Figure 1A). Of note, the Foxa2^iCre^ knock-in mouse expresses Cre recombinase at high levels in notochord starting from E9.5 [19].

Mutant mice in which HIF-1α had been conditionally inactivated in the NP were born with the expected mendelian frequency, reached adulthood and, macroscopically, did not exhibit any obvious phenotypic difference in comparison to control mice. Foxa2^iCre^;HIF-1α^f/+^ and HIF-1α^f/f^ control mice displayed the same histological features and the same pattern of expression of the various markers assessed in this study. Therefore, they were indistinctly used and designated as control mice throughout the study.

Immunohistochemistry for HIF-1α showed that control NP cells accumulate HIF-1α protein in their nuclei (as indicated by black arrows), whereas no positive nuclear signal for HIF-1α (as indicated by white arrows) was detectable in the NP cells of mutant mice at E15.5 (Figure 1B). Conversely, HIF-1α was well expressed in the nuclei of VB cells of both control and mutant specimens (Figure 1C). These data demonstrate that Cre expressed under the control of Foxa2 specifically deleted HIF-1α in the NP.

### Histological analysis of mutant NP upon loss of HIF-1α in the notochord

**Figure 2:**
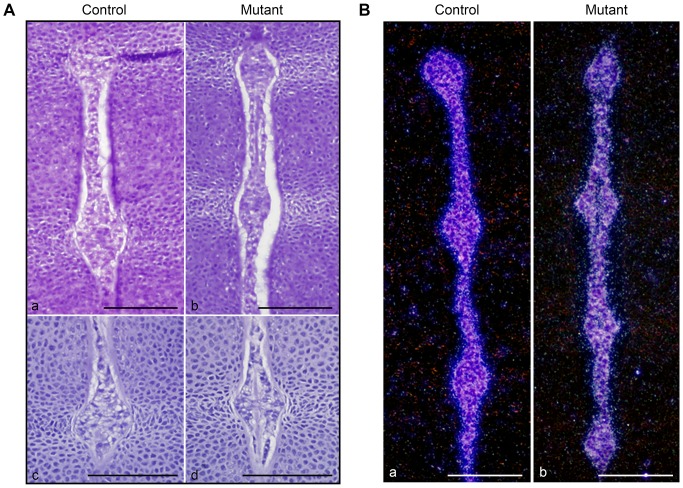
Normal differentiation of the notochord in absence of HIF-1α. **A**. H&E staining of E13.5 spine in control (Foxa2^iCre^;HIF-1α^f/+^) (a) and mutant (Foxa2^iCre^;HIF-1α^f/f^) (b) mice. High magnification of E13.5 NP in control (Foxa2^iCre^;HIF-1α^f/+^) (c) and mutant (Foxa2^iCre^;HIF-1α^f/f^) (d) mice. Bar = 50 µm. **B**. *In situ* hybridization for brachyury mRNA in control (HIF-1α^f/f^) (a) and mutant (Foxa2^iCre^;HIF-1α^f/f^) (b) spine at E13.5. Darkfield pictures are shown. Bar = 50 µm.

As evidenced by H&E staining at E13.5, both control and mutant notochords exhibited similar morphologic features (Figure 2A). Moreover, they displayed undistinguishable patterns of expression of brachyury mRNA, which is a classical notochordal marker (Figure 2B). Therefore, notochord development did not appear to be altered by loss of HIF-1α.

**Figure 3:**
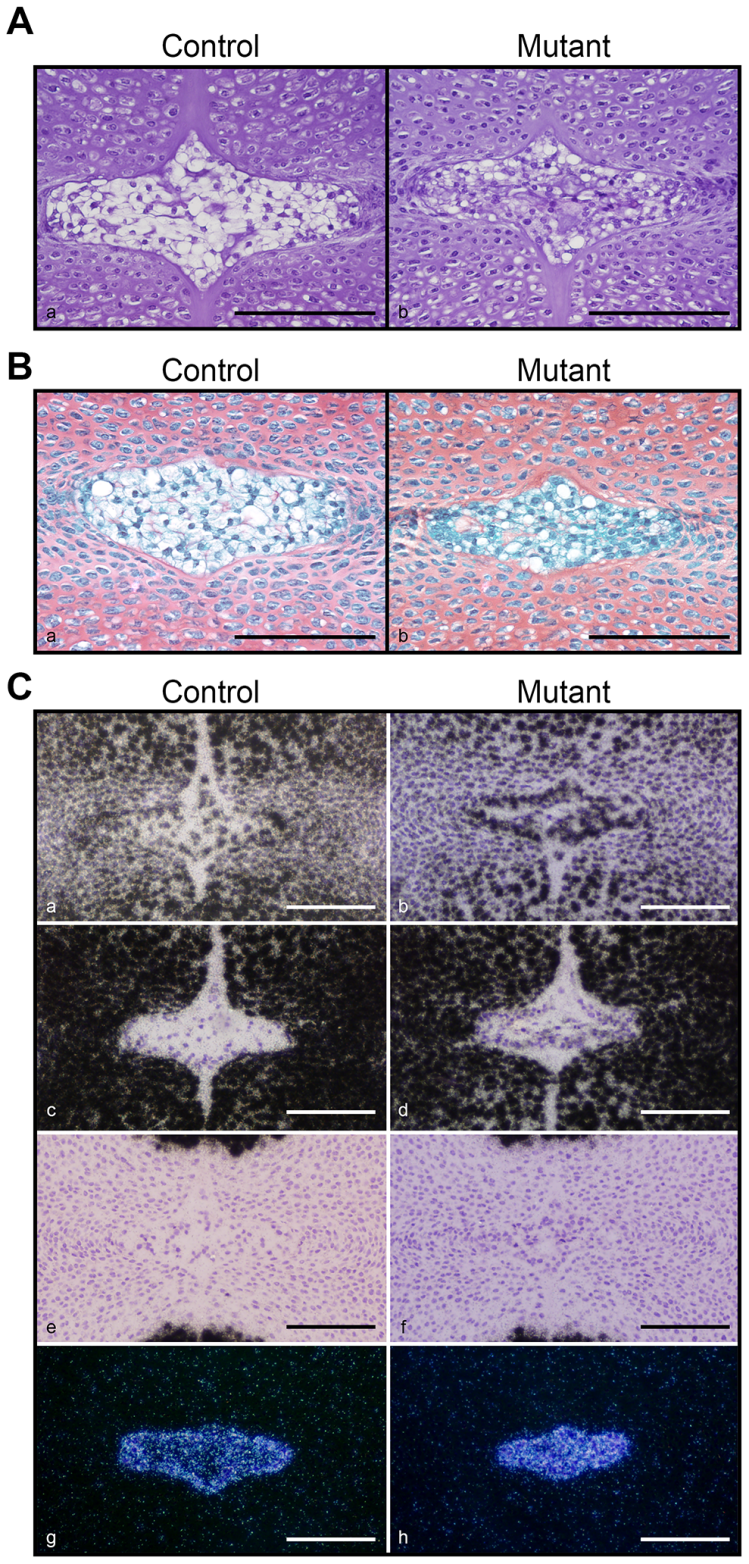
Morphological abnormalities of the mutant NP at E15.5. **A**. H&E staining of E15.5 NP in control (Foxa2^iCre^;HIF-1α^f/+^) (a) and mutant (Foxa2^iCre^;HIF-1α^f/f^) (b) mice. Bar = 50 µm. **B**. Safranin O staining of E15.5 NP in control (HIF-1α^f/f^) (a) and mutant (Foxa2^iCre^;HIF-1α^f/f^) (b) specimens. Bar = 50 µm. **C**. In *situ* hybridization for aggrecan (a,b), collagen II (c,d), collagen X (e,f), and brachyury (g,h) mRNAs in control (HIF-1α^f/f^) (a,c,e,g) and mutant (Foxa2^iCre^;HIF-1α^f/f^) (b,d,f,h) NP at E15.5. Brightfield (a-f) and darkfield (g,h) pictures are shown. Bar = 50 µm.

At E15.5, the mutant NP looked smaller than control, as shown by routine histology (Figure 3A). In addition, mutant NP cells lacked the big vacuoles that are typical of normal NP cells (Figure 3A). We thus asked the question whether the abnormal morphology of mutant NP cells at E15.5 was the consequence of their transdifferentiation into chondrocytes. For this purpose, Safranin O staining and *in situ* hybridizations for detection of brachyury, aggrecan, collagen II and X mRNAs were performed on E15.5 specimens. Brachyury is a marker for notochordal cells. Aggrecan and collagen II are classical chondrocyte markers. Collagen X expression is specific of hypertrophic chondrocytes. The use of these two techniques revealed no detectable differences between controls and mutants. In particular, Safranin O staining confirmed the absence of sulfated glycosaminoglycans (GAG) in NP of both groups (Figure 3B) as previously described [6], [22]. Aggrecan mRNA was detectable both in control and mutant NP (Figure 3C a-b), whereas no convincing evidence for collagen II expression could be found in the NP of either specimen (Figure 3C c-d). Similarly, collagen X mRNA was not expressed in either control or mutant NP (Figure 3C e-f). Conversely, the mRNA encoding for the transcription factor brachyury was present in both (Figure 3C g-h). Of note, aggrecan and collagen II mRNAs were abundantly expressed in the AF and VB of both controls and mutants. Collagen X mRNA expression was restricted to the central hypertrophic region of the VB in both control and mutant specimens (Figure S1). Conversely, as expected, brachyury mRNA expression was absent in both AF and VB compartments (data not shown). We were thus able to confirm that cells of the NP are phenotypically different from cells forming the developing AF and VB. More importantly, we provided clear evidence that mutant NP cells had not acquired a chondrocytic phenotype at E15.5, though loss of HIF-1α as altered their morphology.

**Figure 4:**
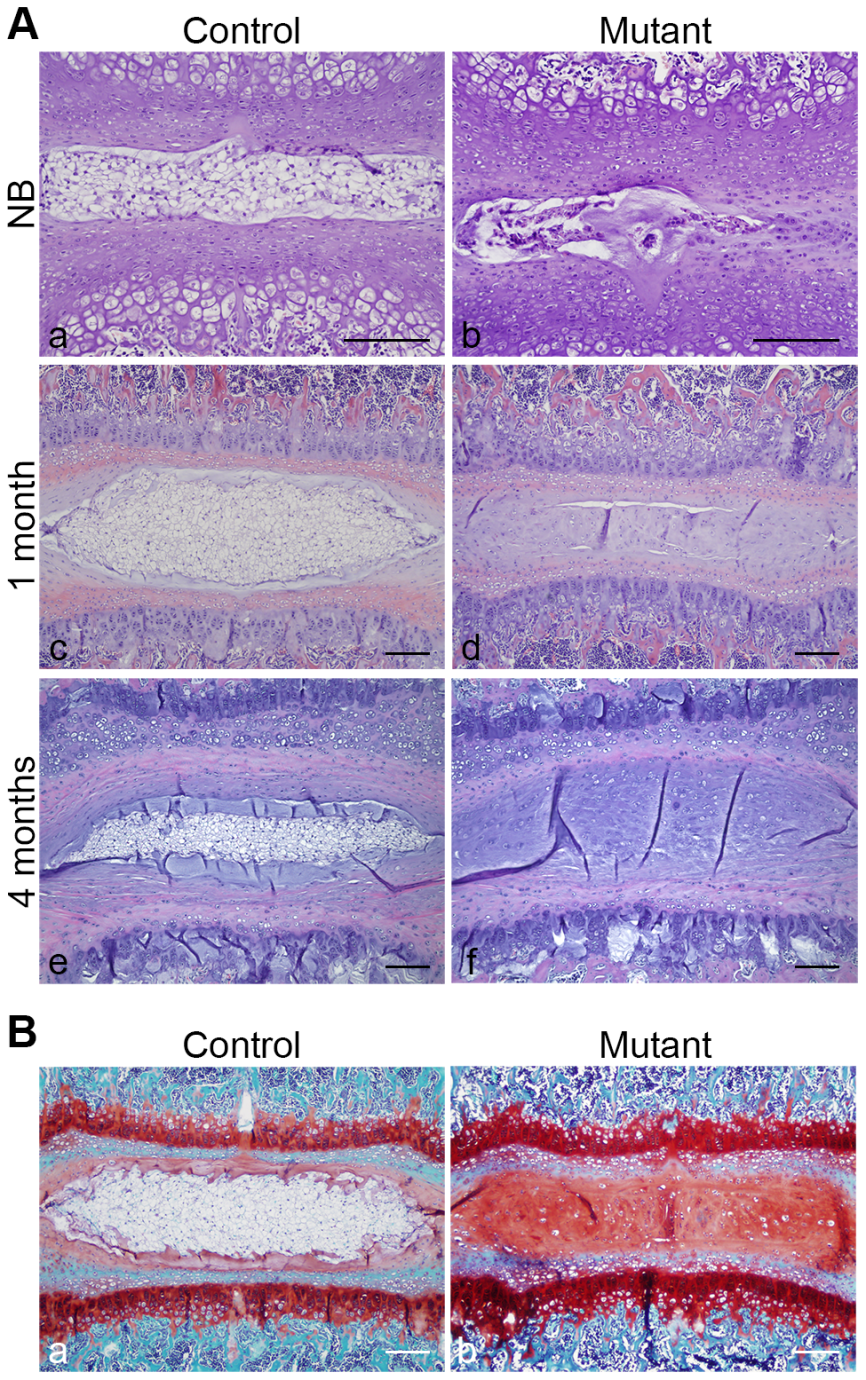
Progressive disappearance of the mutant NP postnatally. **A**. H&E staining of NB (a,b), 1 month (c,d) and 4 months (e,f) NP in control (Foxa2^iCre^;HIF-1α^f/+^) (a,c,e) and mutant (Foxa2^iCre^;HIF-1α^f/f^) (b,d,f) mice. Bar = 50 µm. **B**. Safranin O staining of 1 month (a,b) NP in control (HIF-1α^f/f^) (a) and mutant (Foxa2^iCre^;HIF-1α^f/f^) (b) mice. Bar = 100 µm.

The mutant NP phenotype was more severe at birth (Figure 4A a-b), and by 1 month the NP had virtually disappeared in mutant mice (Figure 4A c-f). In addition, the space previously occupied by the NP was now filled by a fibrocartilaginous tissue that strongly stained for Safranin O (Figure 4B), thus resembling the inner layer of AF. Interestingly, whole mount Alizarin Red S/Alcian Blue staining performed at birth (Figure S2A) and H&E staining of specimens collected between E15.5 and 4 months of age (Figure S2B) did not reveal any obvious abnormality in either mutant VB, AF or CEP, when compared to controls.

**Figure 5:**
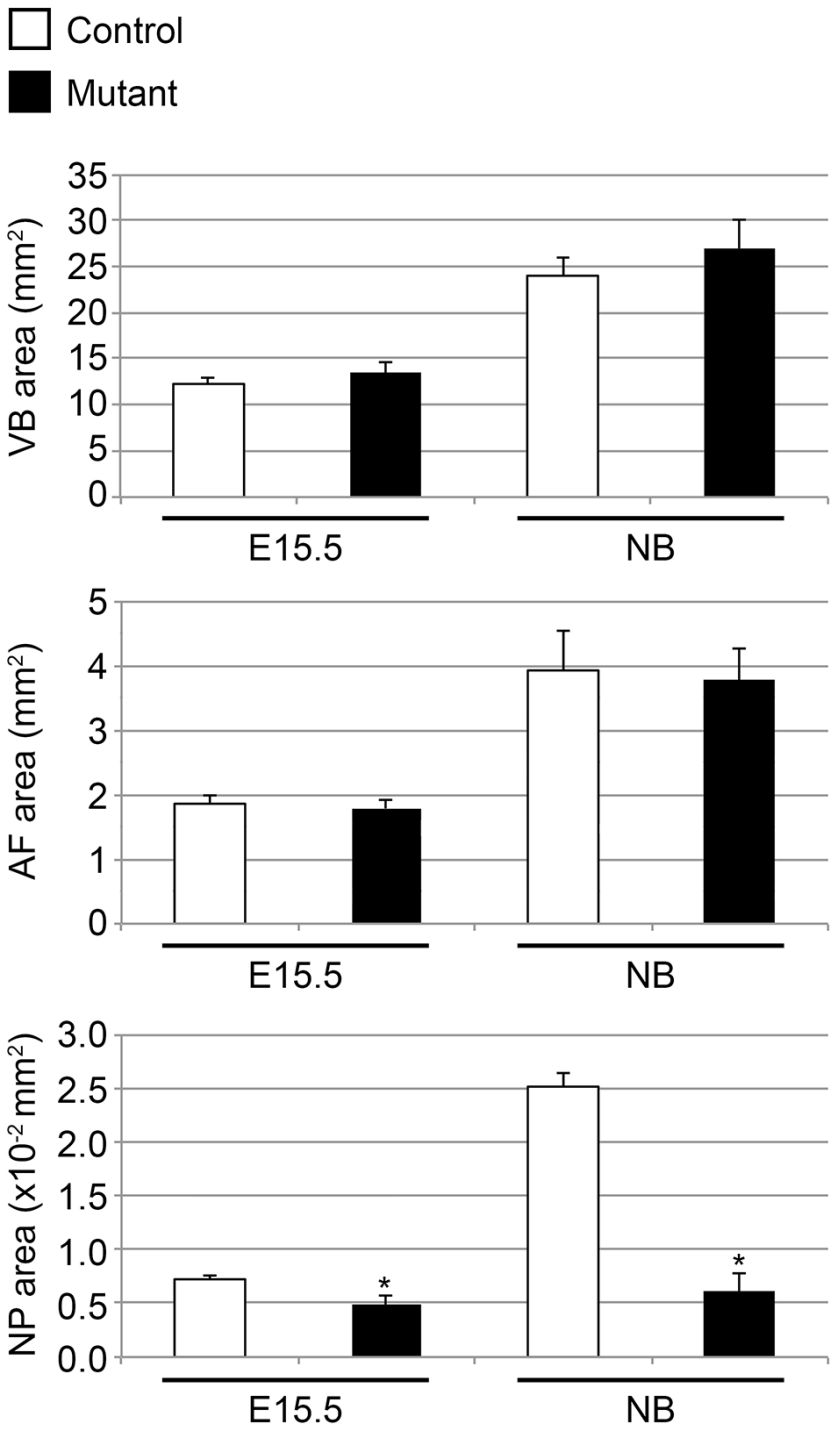
Histomorphometric analysis of control and mutant NP, VB and AF at E15.5 and at birth. Surface measurements of VB, AF and NP of control (white bars) and mutant (black bars) mice at E15.5 and birth. Statistical analysis was performed using the Student's t test. Differences with a p-value <0.05 were considered as statistically significant.

In agreement with these observations, careful histomorphometric analysis confirmed that VB and AF developed indeed normally in mutant mice, with a characteristic 2-fold expansion of their respective surface between E15.5 and birth. On the other hand, as early as E15.5, a significant difference in NP area was detected between control and mutant specimens, and this difference dramatically worsened at birth (Figure 5). In particular, whereas control NP area underwent a 3.5-fold size increase between E15.5 and birth, mutant NP surface barely broadened during the same period of time.

**Figure 6:**
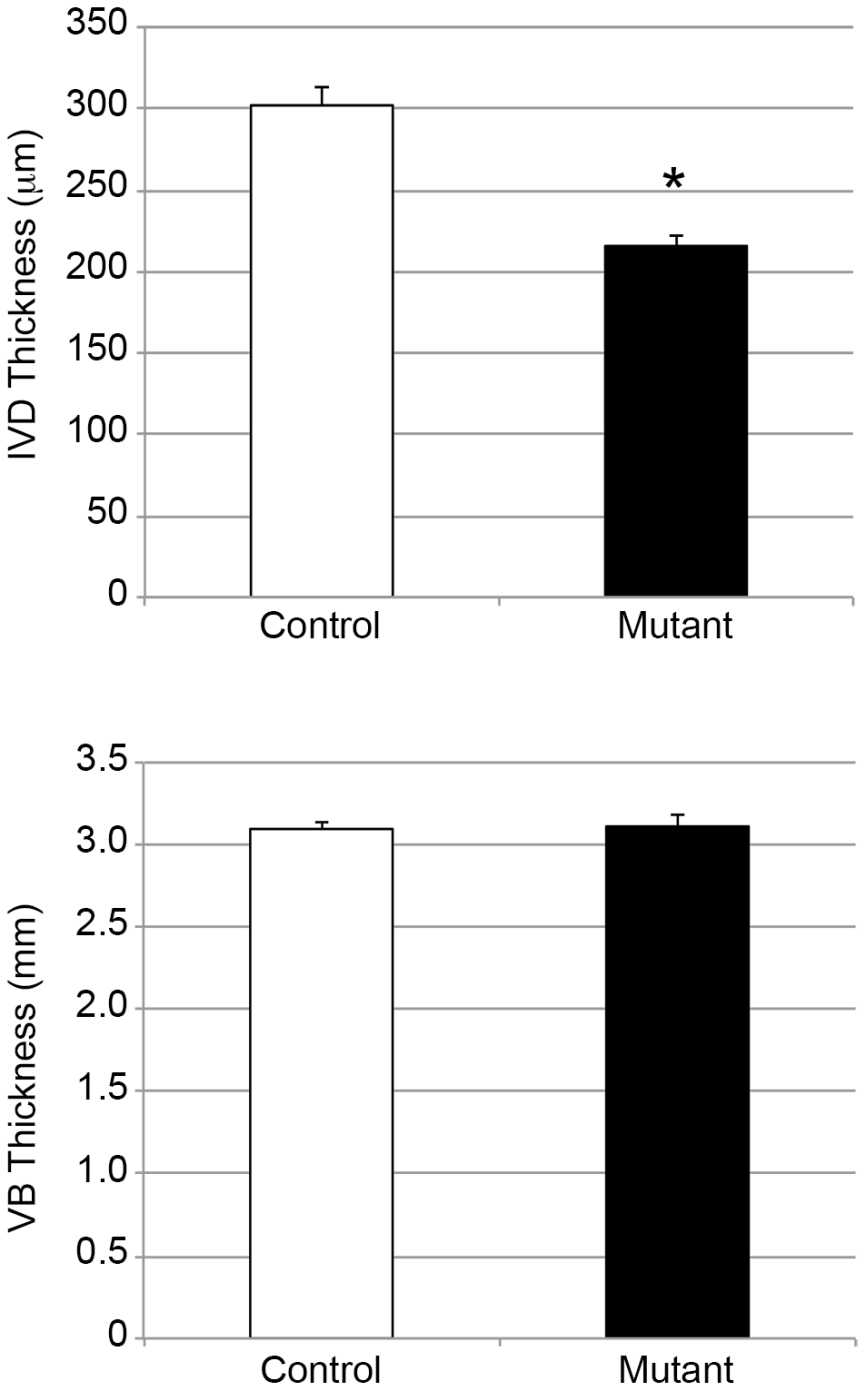
Quantitative analysis of IVD thickness assessed by X rays at 2 months. Measurements of IVD and VB thicknesses of control (white bars) and mutant (black bars) mice at 2 months. Statistical analysis was performed using the Student's t test. Differences with a p-value <0.05 were considered as statistically significant.

At adult age (2 month old), X-rays analysis revealed a significant decrease of IVD height in mutant specimens when compared to controls, whereas VB thickness was not affected (Figure 6).

Taken together, our data indicate that loss of HIF-1α in the cells of the notochordal lineage led to the progressive and, at last, complete disappearance of the NP and to its replacement by fibrocartilage, without, however, altering VB and AF development. Of note, the disappearance of NP led to a reduction of the IVD thickness, which is a major hallmark of IVD degeneration [23].

### Biomechanical properties of the intervertebral disc lacking HIF-1α in the NP

**Figure 7:**
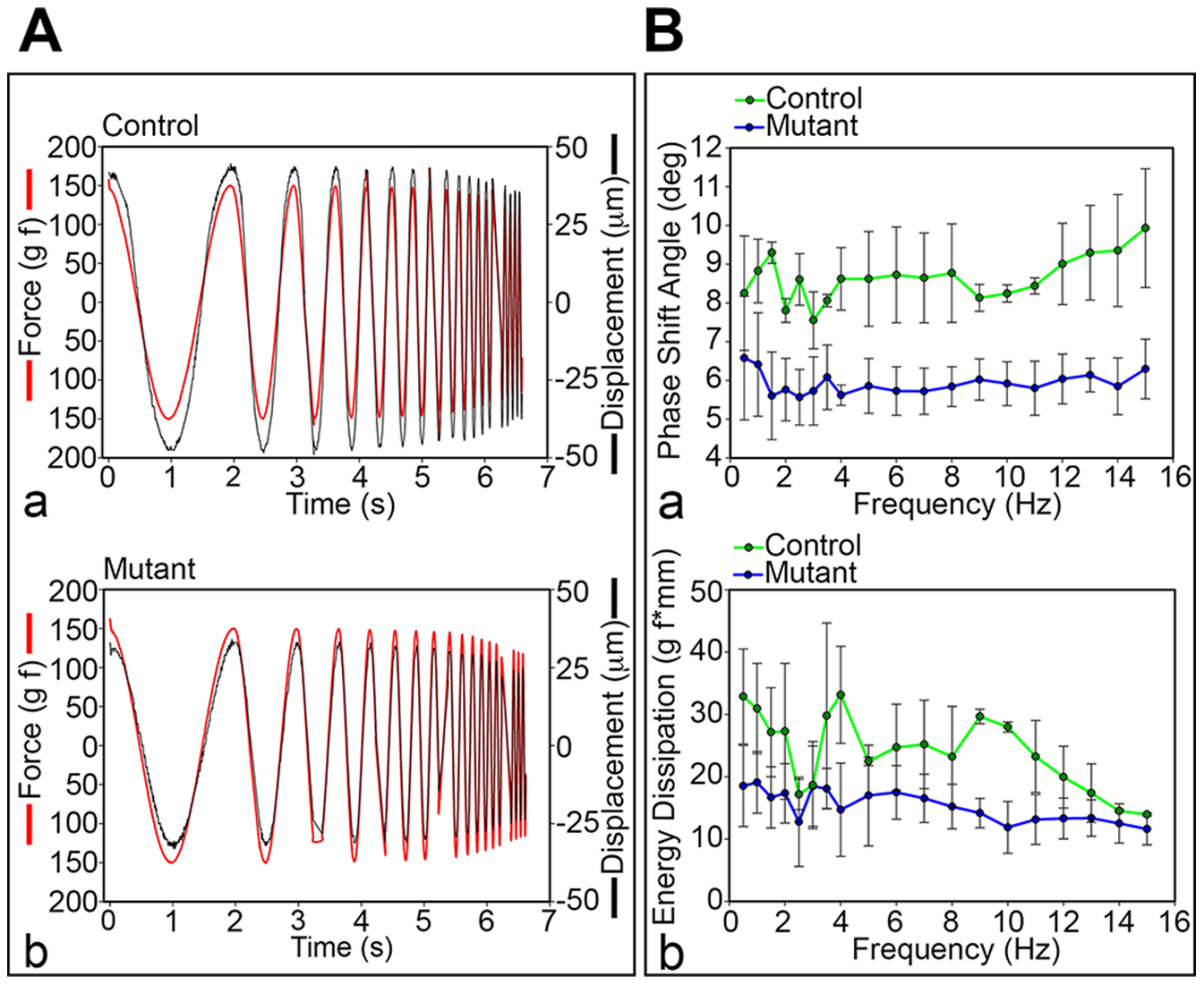
Altered biomechanical properties of the mutant IVD lacking the NP. **A**. Load damping capacity in control (HIF-1α^f/f^) (a) and mutant (Foxa2^iCre^;HIF-1α^f/f^) (b) IVD at 4 months. **B**. Phase shift angle (a) and energy dissipation (b) based on the load damping test in control (HIF-1α^f/f^) and mutant (Foxa2^iCre^;HIF-1α^f/f^) IVD at 4 months. Statistical analysis performed between control and mutant groups: rmANOVA p<0.001 (a) and p<0.05 (b).

To evaluate the impact of the NP loss on the IVD function, biomechanical analyses were performed on spinal motion segments of 4 month-old mice. Dynamic loading of the motion segments isolated from 4 months old mice at ±1.5 N resulted in ∼120–200 µm displacement in each direction (Figure 7A). Calculation of the mean phase shift angle across genotypes at each of the 19 frequencies tested revealed no significant frequency effect on phase shift angle (Figure 7B a). However, comparison between genotypes revealed a ∼40% reduction in phase shift angle among the mutant mice, compared with the controls (rmANOVA, p<0.001). The reduction in phase shift angle in mutant mice suggests that the load damping capacity of the spine was significantly reduced by the HIF-1α deletion. Energy dissipation, calculated from the area within the force-displacement loops, followed a similar but less-pronounced trend as was observed for phase shift angle (Figure 7B b). Again, no frequency effect was detected across genotypes, but there was a significant reduction in energy dissipation among the mutant mice, compared to the HIF-1α^f/f^ mice (rmANOVA, p<0.05). The reduction in energy dissipation in the mice for which HIF-1α has been deleted in the notochordal lineage suggests that the mutant disc has impaired ability to absorb axial energy transmitted across it.

### Lineage analysis of normal and HIF-1α deficient NP cells

**Figure 8:**
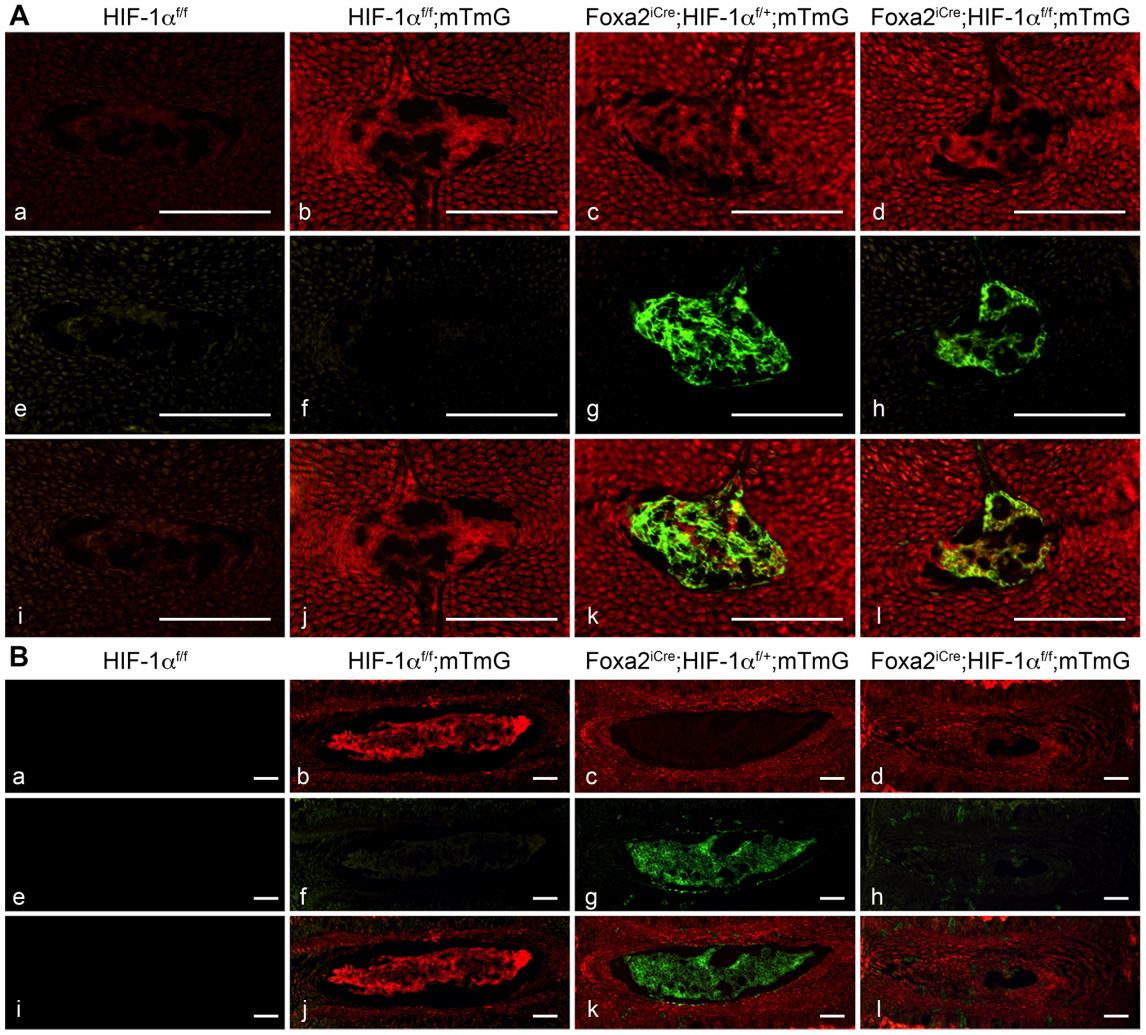
Lineage study in control and mutant IVDs. **A,B**. Detection of fluorescence in frozen sections of NP isolated from E15.5 (A) and 1 month (B) HIF-1α^f/f^ (a,e,i), HIF-1α^f/f^;mTmG (b,f,j), Foxa2^iCre^;HIF-1α^f/+^;mTmG (c,g,k) and Foxa2^iCre^;HIF-1α^f/f^;mTmG (d,h,l) mice, respectively. Red fluorescence (a-d), green fluorescence (e-h) and merged filters (i-l) are shown. Bar = 100 µm.

In order to unequivocally prove that mutant NP cells truly disappear postnatally, we performed a lineage study by using the mT/mG reporter mouse [18]. These mice have *loxP* sites on either side of a membrane-targeted tandem dimer Tomato (mT) cassette, and express red fluorescence in all tissues. When crossed with Cre positive mice, the mT cassette is deleted, allowing expression of the membrane-targeted EGFP (mG) cassette located downstream, in the tissues where Cre has been recombining the reporter gene sequence, whereas red fluorescent protein is no longer synthesized. At E15.5, no auto-fluorescence was detected using either red or green filters in HIF-1α^f/f^ NP (Figure 8A a, e, i). Consistent with the lack of Cre activity, E15.5 HIF-1α^f/f^;mTmG NPs were positive only for the red fluorescent signal (Figure 8A b, f, j). Conversely, NPs from E15.5 Foxa2^iCre^;HIF-1α^f/+^;mTmG displayed both red and green signals, which indicates that a mixed population of recombined and non-recombined cells was indeed present in the normal developing NP (Figure 8A c, g, k). Likewise, also the NPs of E15.5 Foxa2^iCre^;HIF-1α^f/f^;mTmG mutant mice appeared to be composed of a mixed population of recombined and non-recombined cells (Figure 8A d, h, l).

In agreement with the findings at E15.5, at 1 month, no fluorescent signal could be detected in HIF-1α^f/f^, whereas HIF-1α^f/f^;mTmG mice exhibited red fluorescent signal in all the studied tissues (Figure 8B a, e, i and b, f, j). Differently from what we had observed at E15.5, 1 month-old Foxa2^iCre^;HIF-1α^f/+^;mTmG NPs displayed only green fluorescence with no detectable red signal, which indicates that at this time point the population contributing to the normal NPs was exclusively formed by recombined cells (Figure 8B c, g, k). Strikingly, at the same age the fibrocartilaginous tissue that had replaced the NP in Foxa2^iCre^;HIF-1α^f/f^;mTmG mice did not display any green fluorescent signal, which provided unequivocal evidence that all recombined cells had virtually disappeared in the mutant NPs (Figure 8B, d, h, l). These data clearly indicate that at 1 month of age HIF-1α null cells had been completely replaced by a cell population belonging to a lineage distinct from the notochordal one.

### Proliferation and survival of NP cells lacking HIF-1α

**Figure 9:**
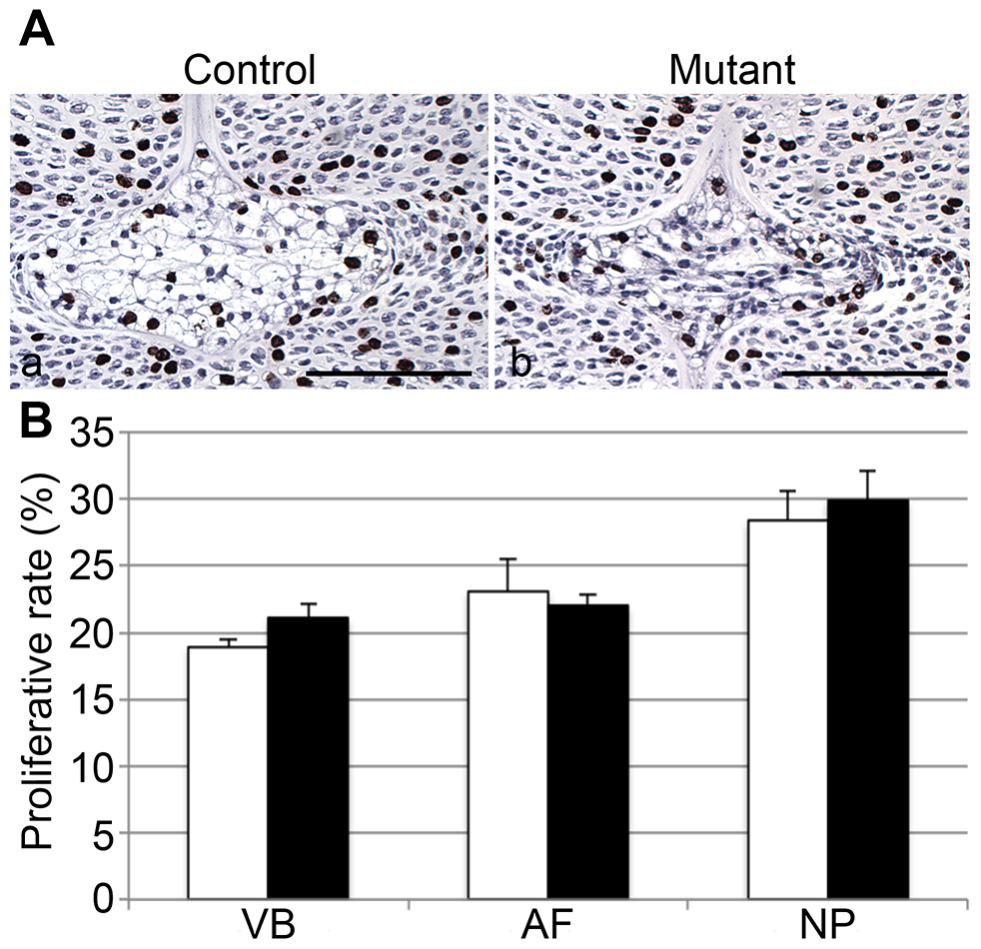
No impaired proliferation of mutant NP cells. **A**. BrdU staining of E15.5 NP in control (Foxa2^iCre^;HIF-1α^f/+^) (a) and mutant (Foxa2^iCre^;HIF-1α^f/f^) (b) mice, respectively. Bar = 50 µm. **B**. Quantification of BrdU staining in VB, AF, NP of control (white bars) and mutant (black bars) specimens.

In order to understand why mutant NP cells disappeared, we then asked the question whether loss of HIF-1α affected their proliferation rate or their survival or both. For this purpose, we performed BrdU assay at E15.5 in mutant and control mice. Notably, proliferation rate was not significantly different between mutant and control NP cells (Figure 9). This finding suggests that both the reduced size of the mutant NP at E15.5 and its postnatal disappearance were not caused by impaired cell proliferation.

**Figure 10:**
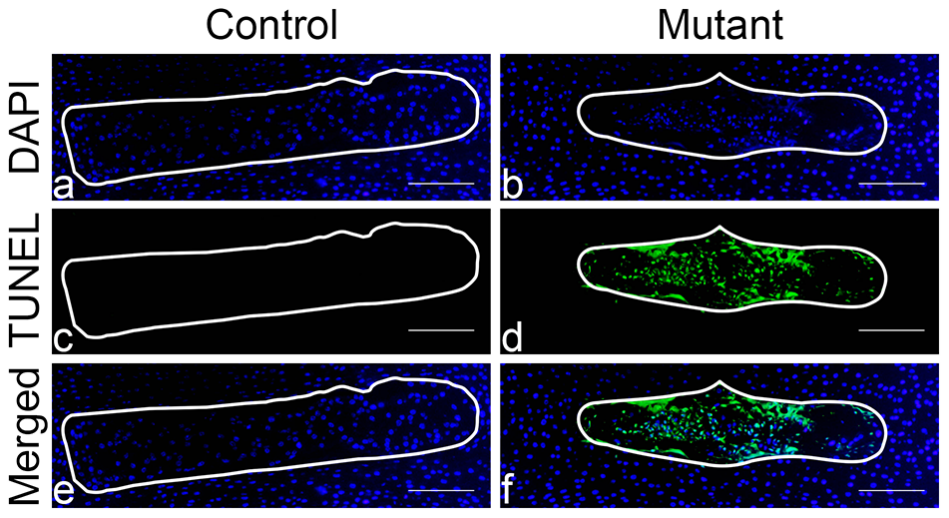
Massive cell death in the mutant NP at birth. Tunel assay of NP at birth in control (Foxa2^iCre^;HIF-1α^f/+^) (a,c,e) and mutant (Foxa2^iCre^;HIF-1α^f/f^) (b,d,f) mice, respectively. DAPI (a,b), TUNEL (c,d) and merged (e, f) microphotographs are presented. Bar = 200 µm.

Next, we evaluated cell death by TUNEL staining. Massive cell death (39.2±4.46%) occurred at birth in the mutant NP, whereas no positive TUNEL signal could be detected in the NP of control specimens (Figure 10 and Figure S3). Conversely, no positive TUNEL signal was present at E15.5 and postnatally in either control or mutant specimens (Figure S4).

These data indicate that cell death is the key pathogenetic factor involved in the postnatal disappearance of the mutant NP. However, cell death is not responsible for the reduced size of the mutant NP at E15.5, which is most likely the consequence of the reduced size of the mutant cells secondary to the lack of their typical big vacuoles.

**Figure 11:**
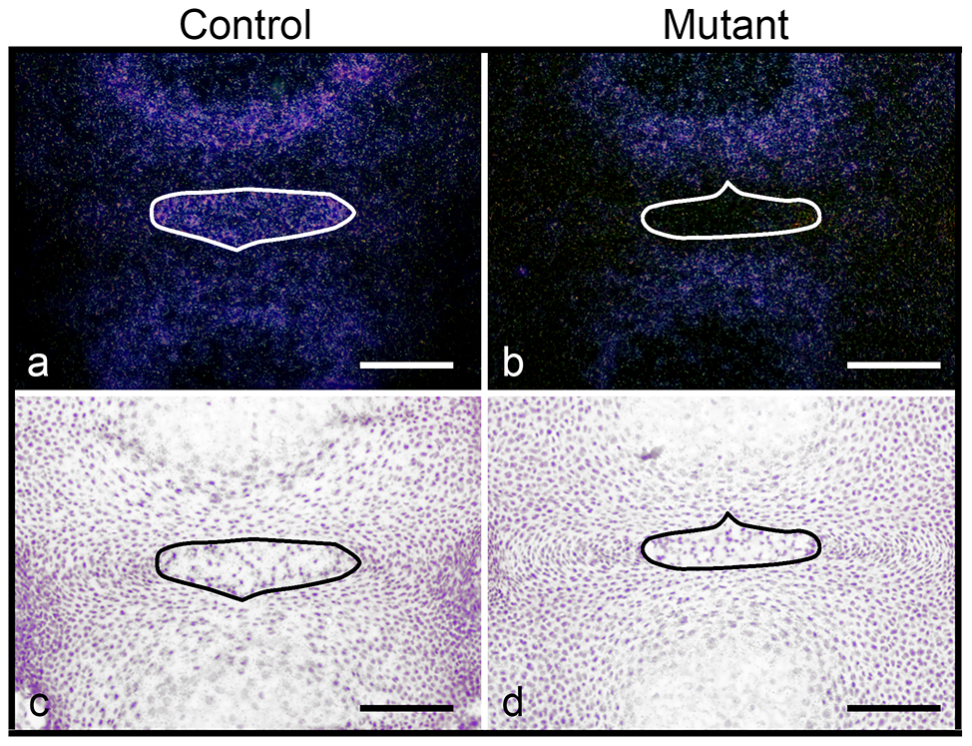
Metabolic changes in the mutant NP. In *situ* hybridization for PGK mRNA in the NP of control (HIF-1α^f/f^) (a,c) and mutant (Foxa2^iCre^;HIF-1α^f/f^) (b,d) mice at E15.5. Darkfield (a,b) and brightfield (c,d) pictures are shown. Bar = 50 µm.

Last, we asked why mutant NP cells died. *In situ* hybridizations showed that phosphoglycerate kinase-1 (PGK) mRNA is highly expressed in the normal NP, consistent with the notion that, likewise the avascular fetal growth plate, the avascular NP lives on anaerobic glycolysis rather than on oxidative phosphorylation. PGK is a known downstream target of HIF-1α, and it catalyzes the reversible transfer of a phosphate group from 1,3-bisphosphoglycerate to ADP producing 3-phosphoglycerate and ATP during glycolysis. Notably, expression of PGK mRNA was not anymore detectable in the NP of mice deficient for HIF-1α, though, as expected was still present in their mutant VBs (Figure 11). Taken together, these findings confirmed that HIF-1α had been efficiently deleted in notochordal cells at E15.5 and suggest that changes in glycolytic metabolism may contribute to the cell death observed in the mutant NP.

## Discussion

Previous studies have shown that the NP is avascular; therefore it is reasonable to assume that NP cells experience oxygen and nutrient deprivation [13], [24]. As HIF-1α is known to be a critical mediator in cellular adaptation to stresses, we decided to focus our attention on its role in the development and homeostasis of the NP.

In this study, we provide unequivocal evidence that HIF-1α is indispensable for development of the NP, since in its absence the NP virtually disappears and is replaced by a fibrocartilaginous structure that resembles the inner layer of the AF. Moreover, our findings indicate that HIF-1α plays a pivotal role in NP biology by finely tuning essential cellular functions such as differentiation and survival. We first demonstrated by immunohistochemistry that HIF-1α expression was efficiently and specifically deleted in NP cells. Next, we performed histological analysis, which unveiled morphologic changes in mutant NP cells as early as E15.5 followed, postnatally, by the progressive disappearance and replacement of the NP with fibrocartilage. This substitutive tissue contained chondrocytic cells synthesizing a matrix that closely resembled the one forming the inner layer of the AF. Notably, these findings are similar to the changes that usually occur in human disc degeneration [25]–[28]. Strikingly, lineage studies and TUNEL assay unequivocally proved that NP cells did not transdifferentiate into chondrocyte-like cells but they rather underwent massive cell death, and were completely replaced by a cell population belonging to a lineage distinct from the notochordal one. Last, loss of the NP in mutant mice significantly reduced the IVD biomechanical properties by decreasing its ability to absorb mechanical stress.

Others and we have previously reported that HIF-1α is a survival and differentiation factor in the context of endochondral bone development [16], . Notably, as it occurs in fetal cartilage, during development of the NP, loss of HIF-1α first affects the morphology of the mutant NP cells, and next causes their massive death, which ultimately leads to the disappearance of the NP. It has been previoulsy demonstrated that HIF-1α plays a pivotal role in adaptative response to stress by regulating proliferation, matrix synthesis and metabolic functions [9], [29], [30]. More particularly, PGK-1 a glycolytic enzyme involved in ATP generation has been identified as a direct downstream target of HIF-1α [16], [31]–[34]. It seems therefore reasonable to assume that HIF-1α deletion in the notochord could impair NP cells ability to maintain a sufficient ATP level in order to survive. Since both the fetal growth plate and the NP are avascular structures, our findings strengthen the notion that HIF-1α is indeed a crucial differentiation and survival factor for avascular tissues during development.

Even if it has been reported that Foxa2^iCre^ is highly expressed in the notochord as early as E9.5 [19], our data indicate that Cre activity is not uniform in the fetal NP, as red fluorescent cells are still present in the E15.5 NP of mice carrying both the Cre and the mT/mG reporter cassette. This lack of uniformity could be secondary to low efficiency of Cre activity; alternatively, it could indicate that the population contributing to the NP is indeed heterogeneous, and that not all the cells forming this tissue are of notochordal origin, as also suggested by previous studies [35]. Interestingly, however, at 1 month of age all the cells forming the normal NP undergo recombination of the mT/mG cassette in presence of Cre, which indicates that, differently from what we observe in fetal life, either partial efficiency of Cre recombinase activity or heterogeneity of the NP population does not become a confounding issue at postnatal time points. Of note, a low efficiency of Cre recombinase activity could explain, in part, why notochord formation remains unaffected by loss of HIF-1α in Foxa2^iCre^;HIF-1α^f/f^ mutant mice, and why the mutant NP cells start disappearing only at birth. Otherwise, it is possible that HIF-1α becomes important for development and homeostasis of the NP mainly at later time points, as the size of the NP gradually increases as well as its demand for oxygen, nutrients and growth factors [14].

Cre expression driven by the Foxa2 promoter appears to be highly specific for the NP cells since no green fluorescent signal could be detected in the AF, CEP or VB of either mutant or control mice by using the mT/mG reporter mouse. In agreement with these findings, no obvious morphological or molecular abnormalities were displayed by either AF, CEP or VB in mutant mice. More importantly, the analysis of the Foxa2^iCre^;HIF-1α^f/f^ mutant mice further corroborates the notion that cells of the NP, or at least their vast majority, are of notochordal origin [4], [6], [35].

In order to fully understand the process that leads to IVD degeneration, it is essential to expand our knowledge of the molecular and cellular mechanisms that drive IVD development and homeostasis. Our study strongly suggests that the HIF pathway may be one of the crucial mechanisms in charge of the proper homeostasis and function of the NP. Along these lines, it is important to highlight that IVDs of mutant mice lacking HIF-1α in the NP have diminished biomechanical properties in comparison to control mice.

All in all, our novel mouse model demonstrates that HIF-1α is essential for normal development and homeostasis of the NP, and it raises the intriguing possibility that this transcription factor could be involved in IVD degeneration in humans. Since our novel mouse model is similar to the IVD degeneration process in humans [23], it could represent a valuable tool to identify novel biological targets involved in IVD degeneration, to unveil novel links between biological phenotypes and biomechanical functions in the NP, and to search for novel therapeutical approaches for the treatment of IVD degeneration.

## Supporting Information

Figure S1 **Normal AF and VB in E15.5 mutant mice.** In *situ* hybridization for aggrecan (a,b), collagen II (c,d) and collagen X (e,f) mRNAs in control (HIF-1α^f/f^) (a,c,e) and mutant (Foxa2^iCre^;HIF-1α^f/f^) (b,d,f) AF and VB at E15.5. Brightfield pictures are shown. Bar = 100 µm.
(TIF)

Figure S2 **Normal VB in adult mutant mice.** **A**. Whole mount Alizarin Red S/Alcian Blue staining: skeletal preparations of NB spines in control (Foxa2^iCre^;HIF-1α^f/+^) (a) and mutant (Foxa2^iCre^;HIF-1α^f/f^) (b) mice. **B**. H&E staining of E.15.5 (a,b), NB (c,d), 1 month (e,f) and 4 months (g,h) VB in control (Foxa2^iCre^;HIF-1α^f/+^) (a,c,e,g) and mutant (Foxa2^iCre^;HIF-1α^f/f^) (b,d,f,h) mice, respectively. Bar = 100 µm.
(TIF)

Figure S3 **Tunel assay quantification at birth.** Quantification of cell death is expressed as the percentage of Tunel positive cells over DAPI positive cells at birth. ND stands for not detected in the NP of control specimens.
(TIF)

Figure S4 **Tunel assay.** Tunel assay of NP at E15.5 (a,b), 1 month (c,d) and 4 months (e,f) in control (Foxa2^iCre^;HIF-1α^f/+^) (a,c,e) and mutant (Foxa2^iCre^;HIF-1α^f/f^) (b,d,f) mice, respectively. Bar = 100 µm.
(TIF)
