# Supplementary figures and images for: Loss of HIF-1α in the Notochord Results in Cell Death and Complete Disappearance of the Nucleus Pulposus
Source: PLoS One. 2014 Oct 22;9(10):e110768. doi: 10.1371/journal.pone.0110768 (PMC4206488; doi:10.1371/journal.pone.0110768)

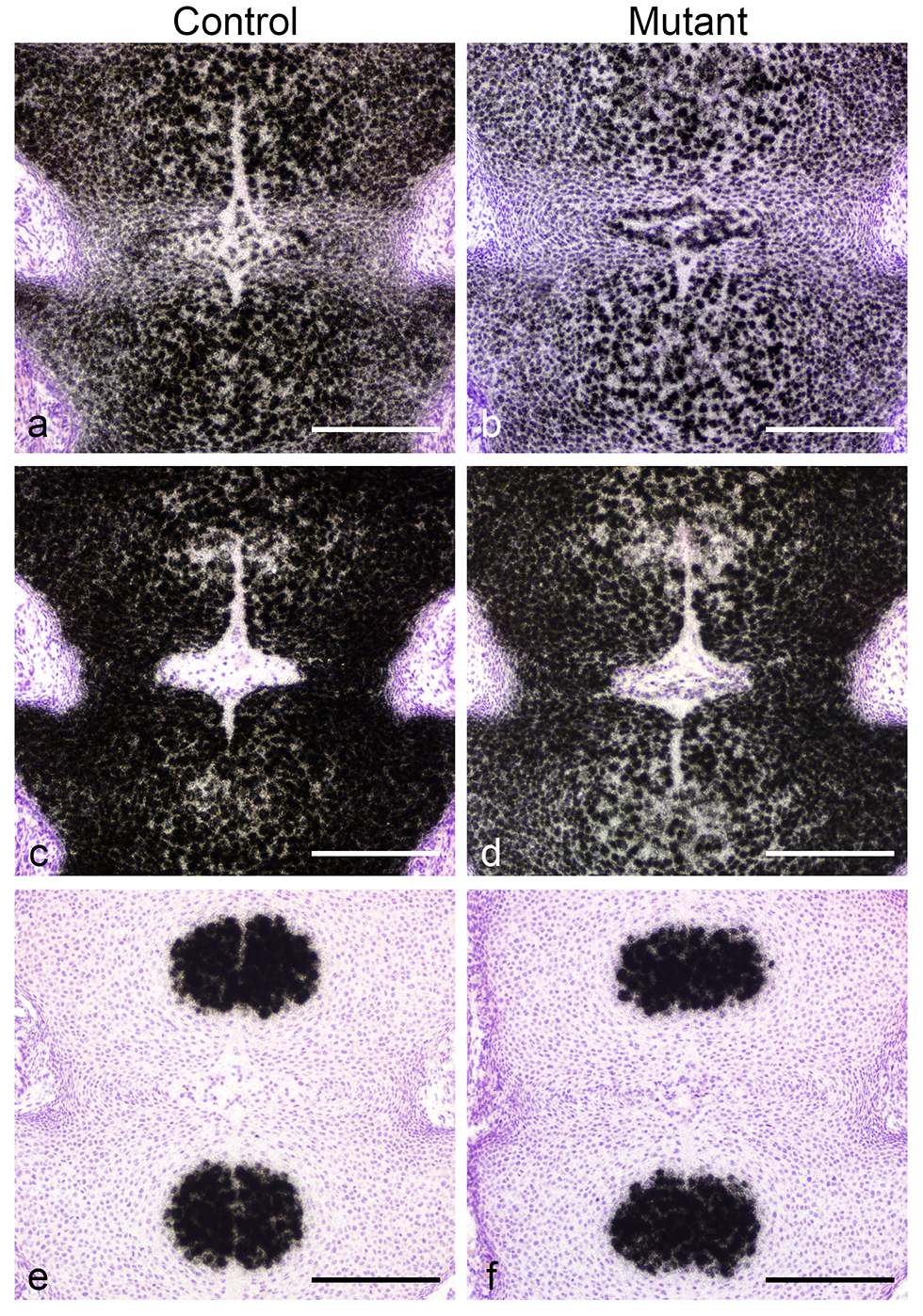

Supplement: Figure S1 — Normal AF and VB in E15.5 mutant mice. In situ hybridization for aggrecan (a,b), collagen II (c,d) and collagen X (e,f) mRNAs in control (HIF-1αf/f) (a,c,e) and mutant (Foxa2iCre;HIF-1αf/f) (b,d,f) AF and VB at E15.5. Brightfield pictures are shown. Bar = 100 µm. (TIF) [file pone.0110768.s001.tif]

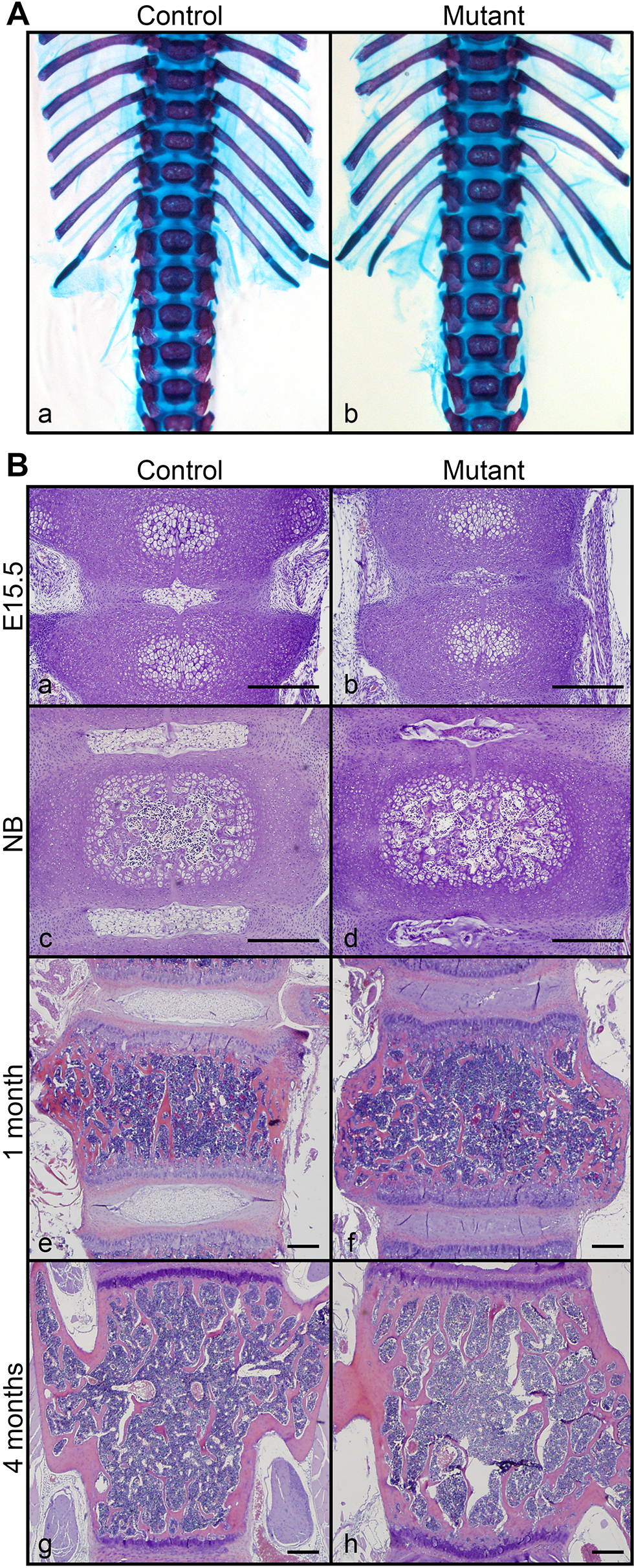

Supplement: Figure S2 — Normal VB in adult mutant mice. A. Whole mount Alizarin Red S/Alcian Blue staining: skeletal preparations of NB spines in control (Foxa2iCre;HIF-1αf/+) (a) and mutant (Foxa2iCre;HIF-1αf/f) (b) mice. B. H&E staining of E.15.5 (a,b), NB (c,d), 1 month (e,f) and 4 months (g,h) VB in control (Foxa2iCre;HIF-1αf/+) (a,c,e,g) and mutant (Foxa2iCre;HIF-1αf/f) (b,d,f,h) mice, respectively. Bar = 100 µm. (TIF) [file pone.0110768.s002.tif]

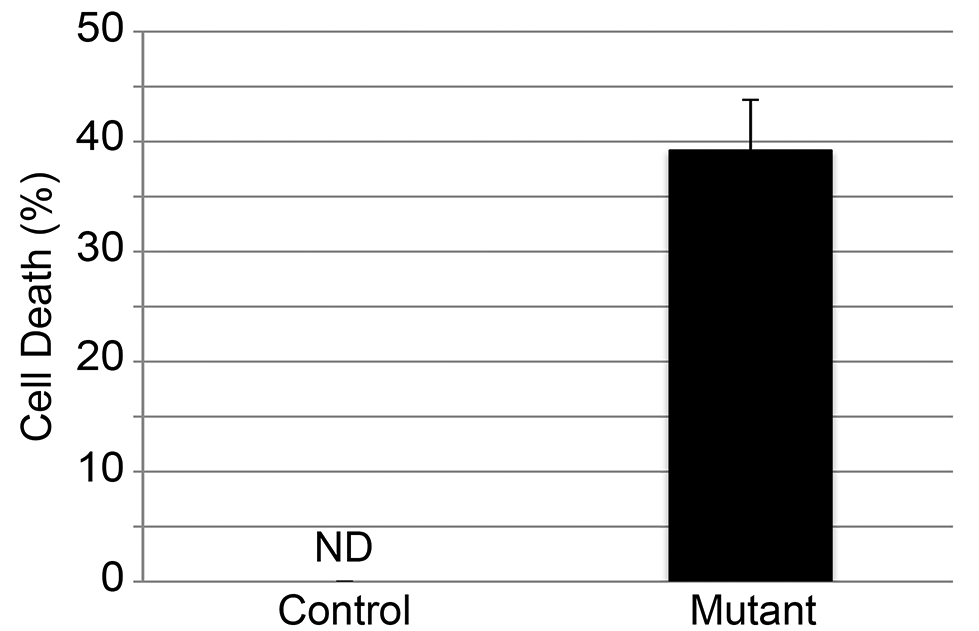

Supplement: Figure S3 — Tunel assay quantification at birth. Quantification of cell death is expressed as the percentage of Tunel positive cells over DAPI positive cells at birth. ND stands for not detected in the NP of control specimens. (TIF) [file pone.0110768.s003.tif]

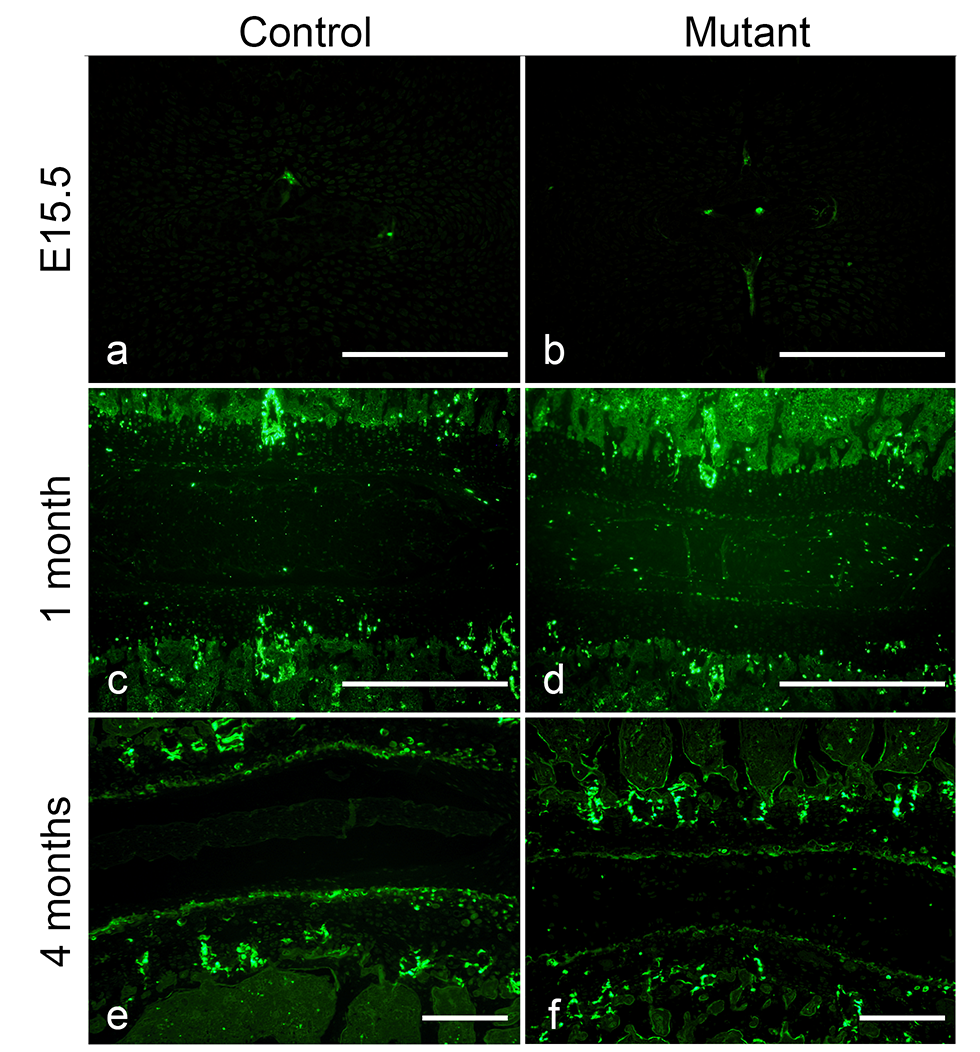

Supplement: Figure S4 — Tunel assay. Tunel assay of NP at E15.5 (a,b), 1 month (c,d) and 4 months (e,f) in control (Foxa2iCre;HIF-1αf/+) (a,c,e) and mutant (Foxa2iCre;HIF-1αf/f) (b,d,f) mice, respectively. Bar = 100 µm. (TIF) [file pone.0110768.s004.tif]
